# Increased prevalence of *Escherichia coli* strains from food carrying *bla*_NDM_ and *mcr-1*-bearing plasmids that structurally resemble those of clinical strains, China, 2015 to 2017

**DOI:** 10.2807/1560-7917.ES.2019.24.13.1800113

**Published:** 2019-03-28

**Authors:** Xiaobo Liu, Shu Geng, Edward Wai-Chi Chan, Sheng Chen

**Affiliations:** 1 Shenzhen Key Lab for Food Biological Safety Control, Food Safety and Technology Research Center, Hong Kong PolyU Shen Zhen Research Institute, Shenzhen, China; 2 College of Food Science and Engineering, Northwest A&F University, Yangling, Shaanxi, China; 3 The State Key Lab of Chirosciences, Department of Applied Biology and Chemical Technology, The Hong Kong Polytechnic University, Hung Hom, Kowloon, Hong Kong SAR

**Keywords:** *bla*_NDM_, *mcr-1*, *E. coli*, food, plasmid resistance

## Abstract

**Introduction:**

Emergence of resistance determinants of *bla*_NDM_ and *mcr-1* has undermined the antimicrobial effectiveness of the last line drugs carbapenems and colistin.

**Aim:**

This work aimed to assess the prevalence of *bla*_NDM_ and *mcr-1* in *E. coli* strains collected from food in Shenzhen, China, during the period 2015 to 2017.

**Methods:**

Multidrug-resistant *E. coli* strains were isolated from food samples. Plasmids encoding *mcr-1* or *bla*_NDM_ genes were characterised and compared with plasmids found in clinical isolates.

**Results:**

Among 1,166 non-repeated cephalosporin-resistant *E. coli* strains isolated from 2,147 food samples, 390 and 42, respectively, were resistant to colistin and meropenem, with five strains being resistant to both agents. The rate of resistance to colistin increased significantly (p < 0.01) from 26% in 2015 to 46% in 2017, and that of meropenem resistance also increased sharply from 0.3% in 2015 to 17% in 2017 (p < 0.01). All meropenem-resistant strains carried a plasmid-borne *bla*_NDM_ gene. Among the colistin-resistant strains, three types of *mcr-1*-bearing plasmids were determined. Plasmid sequencing indicated that these *mcr-1* and *bla*_NDM_-bearing plasmids were structurally similar to those commonly recovered from clinical isolates. Interestingly, both *mcr-1*-bearing and *bla*_NDM_-bearing plasmids were transferrable to *E. coli* strain J53 under selection by meropenem, yet only *mcr-1*-bearing plasmids were transferrable under colistin selection.

**Conclusion:**

These findings might suggest that mobile elements harbouring *mcr-1* and *bla*_NDM_ have been acquired by animal strains and transmitted to our food products, highlighting a need to prevent a spike in the rate of drug resistant food-borne infections.

## Introduction

Antimicrobial resistance poses an increasing risk to human and animal health worldwide. In particular, carbapenem resistance mediated by serine β-lactamases and metallo-β-lactamases (MBLs), such as the OXA enzymes produced by *Acinetobacter baumannii* and *Klebsiella pneumoniae* carbapenemase (KPC-1) and New Delhi metallo-β-lactamase (NDM-1) produced by Enterobacteriaceae, is associated with a high mortality rate among hospitalised patients [1,2]. NDM-1, a type of Ambler class B metallo-β-lactamases (MBLs), exhibits high hydrolytic activity against almost all known β-lactam antimicrobials (except aztreonam), including the last-line carbapenems [3,4]. It was first found to be produced by *K. pneumoniae* and *Escherichia coli* strains isolated from a Swedish patient of Indian origin who was admitted to hospital in New Delhi, India [5]. Thereafter, the *bla*_NDM-1_ gene disseminated in various countries and regions such as China, the Middle East, South East Asia and Europe [4]. This multidrug resistance gene, which may be located on either plasmids or chromosome [3,6,7], leaves few therapeutic options for infected patients. In China, Ho et al. reported the first isolation of *bla*_NDM-1_-positive *E. coli* from a 1-year-old infant and its mother in 2011 [8]. NDM-1-producing Enterobacteriaceae have since disseminated to various provinces in China, with the majority of such strains isolated from stool samples [9]. However, reports of isolation of carbapenem-resistant Enterobacteriaceae (CRE) from food samples remain scarce around the world.

Tigecycline and colistin have been regarded as last-line antibiotics used to treat serious infections caused by carbapenemase-producing Enterobacteriaceae [10]. Colistin (polymyxin E) belongs to the family of polymyxins which act against Gram-negative bacteria, including most species of Enterobacteriaceae, with broad-spectrum activity. Until 2015, almost all reported polymyxin resistance mechanisms were coded on the chromosome and mediated by mutations in two component regulatory systems (PmrAB, PhoPQ), loss of lipopolysaccharide, or *mgrB* inactivation in the case of *K. pneumoniae* [10,11]. Importantly, a report by Liu et al. in November 2015 described the emergence of a conjugative plasmid-mediated colistin resistance gene, *mcr-1*, which encodes an enzyme (MCR-1) that belongs to the phosphoethanolamine transferase enzyme family and leads to altered bacterial lipid A in Enterobacteriaceae through modification of the phosphoethanolamine moiety [10-12]. Identification of *mcr-1* as a plasmid-mediated resistance mechanism highlights its potential to act as a transmissible colistin resistance determinant. Following this discovery, several reports have documented the presence of *mcr-1* in strains of different species of Enterobacteriaceae that exhibited multidrug resistance phenotypes and were recovered from farm animals, food samples, human faecal samples, environmental samples and samples collected in clinical settings [11-14]. Plasmid types including IncX4, IncI2, IncP, IncFII and IncHI2, which harbour the *mcr-1* gene, have been reported [12,14-16].

Several recent studies have reported the recovery of strains that harboured both the *bla*_NDM_ and *mcr-1* genes, including *bla*_NDM-1_/*bla*_NDM-5_/*bla*_NDM-7_ and *mcr-1* in Enterobacteriaceae species, especially in *E. coli* [17-19], as well as *bla*_NDM-9_ and *mcr-1* in *Cronobacter sakazakii* [20]. These reports focused on the epidemiological features, evolutionary origin and dissemination routes of resistance genes in different countries. However, few of these studies involved surveillance of prevalence of the *bla*_NDM_ and *mcr-1* genes for a prolonged period. Here, we report the presence of *bla*_NDM_ and *mcr-1* in *E. coli* isolated from food samples in Shenzhen, China, during the period 2015 to 2017 and assessed the prevalence of both genes in these *E. coli* food isolates. We investigated the genomic structures of the mobile genetic elements that encoded *bla*_NDM_ and/or *mcr-1* in these strains to elucidate the transferability of these important resistance determinants.

## Methods

### Bacterial isolation

*E. coli* isolates were obtained from food samples, including pork, chicken, beef and shrimps, collected from wet markets and supermarkets in Shenzhen, Guangdong Province, China during the period from 10 August 2015 to 17 April 2017. Three isolation methods were employed to increase the yield. Details of these are described in the Supplement. 

### Antimicrobial susceptibility testing

All *E. coli* isolates recovered from food samples were subjected to determination of their antimicrobial susceptibilities using the agar dilution method according to the Clinical and Laboratory Standards Institute (CLSI) except for colistin and tigecycline that was tested using the broth-microdilution method [21]. We used resistance breakpoints published by the CLSI [21]. *E. coli* strain ATCC 25922 was tested as the quality control strain. Further *E. coli* isolates from the same food sample and exhibiting identical MIC profile were considered as similar clones and eliminated from further analysis. Only non-duplicated *E. coli* isolates from each sample were used for further analysis.

### Characterisation of *bla*_NDM_ and *mcr-1* positive *Escherichia coli* strains

All *E. coli* strains were screened for the presence of the *bla*_NDM_ and *mcr-1* gene by PCR using primers as previously described [10,22]. PCR products were purified and subjected to Sanger sequencing to confirm the genetic identity.

Methodological details for the conjugation experiments, *Xba*I-PFGE, S1-PFGE and Southern hybridisation are given in the Supplement. 

### Plasmid sequencing and bioinformatics analyses

Plasmid sequencing was performed as previously described using the Illumina and PacBio RS II platforms [14]. All plasmid sequences were submitted to the RAST tool for annotations and modified manually by BLAST [23]. The Easyfig software was used in comparative plasmid analysis [24].

### Statistical analysis

The statistical analysis was performed by Minitab16 software (Minitab, version 16.2.3, Minitab Inc., State College, United States). The resistance rates of *E. coli* to colistin and meropenem in 2015, 2016 and 2017 were determined by the chi-squared test. The threshold for significant difference was p < 0.05.

## Results

### Prevalence of cephalosporin-resistant *Escherichia coli* in food products

A total of 1,166 (55%) non-repeated cephalosporin-resistant *E. coli* isolates were recovered from 2,137 food samples purchased during the period from 2015 to 2017 in Shenzhen, China, for the Food-borne Pathogen Surveillance Programme conducted by the Shenzhen Key Laboratory for Food Biological Food Safety Control. No food sample was found to contain more than one type of *E. coli* isolate. Among the 2,137 food samples, 1,368 were purchased from wet markets, and 769 samples were purchased from supermarkets of different regions in Shenzhen. These food samples included 1,182 pork, 230 beef, 383 chicken and 342 shrimp samples (Supplementary Table S1). Among the 1,166 ceftaxidime-resistant *E. coli* isolates, 385 (52%) *E. coli* were isolated from 747 food samples in 2015, 570 (56%) from 1,019 food samples in 2016, and 211 (57) from 371 food samples in 2017 (Table 1). Among these *E. coli* isolates, 710 were isolated from the 1,182 pork samples (60%), 107 were recovered from the 230 beef samples (47%), 315 from the 383 chicken samples (82%) and 34 from the 342 shrimp samples (10%). The detailed sampling and isolation information is provided in Supplementary Table S1.

**Table 1:** Isolation rate of *Escherichia coli* strains resistant to ceftaxime, *mcr-1* bearing strains, *bla*_NDM_ bearing strains and strains harbouring both *mcr-1* and *bla*_NDM_ genes, Shenzhen, 2015–2017 (n = 2,137)

Year	Number of food samples	Cefotaxime-resistant *E. coli* isolates										*mcr-1*-bearing *E. coli*		*bla*_NDM_-bearing *E. coli*, with or without *mcr-1*			
		Total	Isolation rate (%)^a^	Pork		Beef		Chicken		Shrimp		Total	*mcr-1* positivity rate (%)^a^	Total	*bla*_NDM_ positivity rate (%)^a^	Number of *mcr-1*-positive strains	*bla*_NDM_/*mcr-1* positivity rate (%)
				n	%	n	%	n	%	n	%						
2015	747	385	52 (A)	248	57	33	46	97	75	7	6	98	25 (B)	1	0.3 (E)	0	0
2016	1,019	570	56 (A)	319	52	61	53	168	86	22	12	207	36 (C)	5	1 (E)	0	0.2
2017	371	211	57 (A)	143	65	13	30	50	88	5	10	97	46 (D)	36	17 (F)	5	2
**Total**	**2,137**	**1,166**	**55**	**710**	**60**	**107**	**47**	**315**	**82**	**34**	**10**	**402**	**34**	**42**	**4**	**5**	**0.4**

^a^ Percentage was calculated by dividing the number of *E. coli* isolates carrying the indicated resistance genes by the total number of cefotaxime-resistant *E. coli* isolates obtained in each year; percentages labelled with the same letter in brackets indicated that no significant difference was observed between two variants; percentages labelled with different letters indicated that a significant difference was observed between two variants (p < 0.05); (B) vs (C): p = 0.000; (C) vs (D): p = 0.014; (B) vs (D): p = 0.000; (B) vs (C) vs (D): p = 0.000; (E) vs (F): p = 0.000.

### Antimicrobial susceptibility of *Escherichia coli* food isolates

We determined the minimal inhibitory concentrations (MIC) of various antibiotics for all 1,166 cephalosporin-resistant *E. coli* isolates. These strains were found to be resistant to most of the antibiotics tested, with a resistance rate of 100% to ampicillin, cefotaxime and ceftriaxone, and resistance to the other agents as follows: 95% to sulfamethoxazole/trimethoprim, 95% to tetracycline, 86% to chloramphenicol, 82% to nalidixic acid, 64% to kanamycin and 59% to ciprofloxacin. The rate of resistance to colistin was ca 33%. On the other hand, these strains were susceptible to tigecycline (100%), amikacin (97%), ceftazidime/avibactam (96%) and meropenem (96%). For the 390 *E. coli* strains that were resistant to colistin, the rate of resistance to different antibiotics was slightly higher than the resistance rate of all *E. coli* isolates, with the of exception of tigecycline (0%), ceftazidime/avibactam (2%) and meropenem (2%) (Supplementary Table S2). Forty-two *E. coli* strains that were resistant to meropenem were also resistant to most of other antibiotics. Five of these 42 strains were also resistant to colistin, but they were mostly susceptible to amikacin (98%) (Supplementary Table S2). Among the 390 colistin-resistant *E. coli* strains, the rate of resistance to colistin increased significantly over the years, with 26% in 2015, 36% in 2016 and 46% in 2017 (p < 0.05). Among the 42 strains that were resistant to meropenem, the rate of meropenem resistance increased significantly from 0.3% in 2015 to 1% in 2016 and 17% in 2017 (p < 0.001) (Table 1). Interestingly, five meropenem-resistant strains were also resistant to colistin, accounting for 0.4% of the total number of *E. coli* strains isolated from food products in this study (Table 1).

### Prevalence of *mcr-1* and *bla*_NDM_ in *Escherichia coli* isolates from food

**Figure 1:**
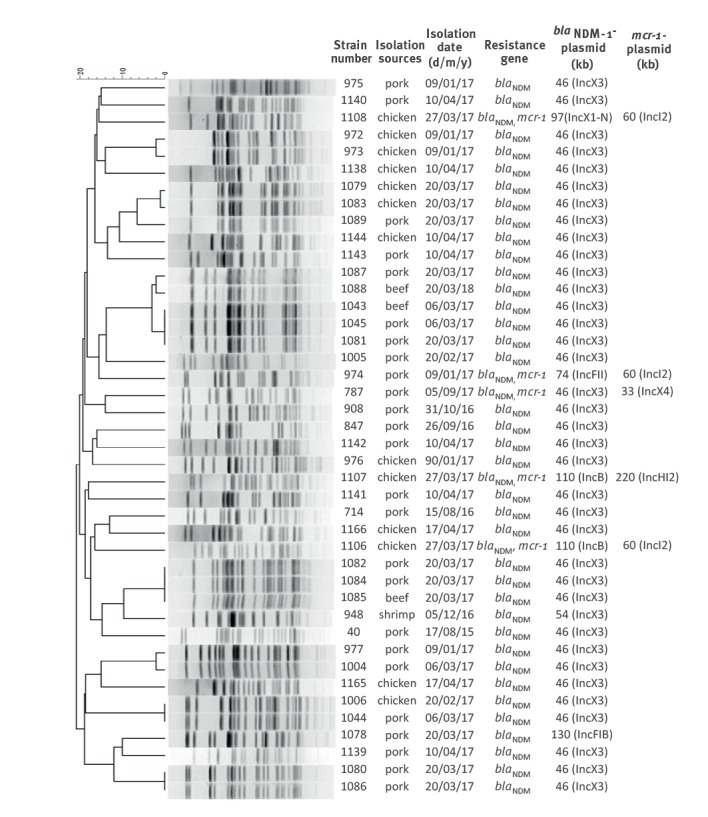
*Xba*I-PFGE pattern of *Escherichia coli* isolates resistant to meropenem, Shenzhen, 2015–2017 (n = 42)

All 390 colistin-resistant *E. coli* strains were subjected to screening for the presence of the *mcr-1*, *mcr-2*, *mcr-3* and *mcr-4* genes; all of them carried the *mcr-1* gene but not the other *mcr* genes. Screening for carbapenemase genes among the 42 meropenem-resistant *E. coli* strains showed that all of them harboured *bla*_NDM-1_ gene except for strains 787, 974, 977, 1079, 1106 and 1107, which were found to harbour the *bla*_NDM-5_ gene. No other carbapenemase genes, such as the *bla*_KPC_, *bla*_VIM_, *bla*_IMP_, *bla*_OXA48_-like genes, could be detected in these strains. Consistently, the five *E. coli* strains that were resistant to both meropenem and colistin carried both *mcr-1* and *bla*_NDM_ genes. Genetic characterisation by PFGE of the 42 *bla*_NDM-1_-carrying *E. coli* strains showed that the majority were genetically unrelated, suggesting that the spread of these strains was mainly due to acquisition of *bla*_NDM_ by different *E. coli* strains, rather than clonal dissemination of strains that had acquired the *bla*_NDM_ gene (Figure 1). Nevertheless, evidence of clonal transmission was also observed; examples include strains 1080/1086 and 972/973, which were isolated from different pork or chicken samples purchased on the same date, as well as strains 1043/1045/1081, 1082/1084/1085, 1006/1044 and 977/1004, which were isolated from different meat products on different dates.

All these plasmids were conjugative plasmids.

### Genetic characterisation of *bla*_NDM_-bearing *Escherichia coli* strains

To investigate the genetic characteristics of *E. coli* strains carrying *bla*_NDM_, conjugation experiments were performed. The meropenem resistance phenotype of all 42 *bla*_NDM_-bearing strains tested was transferrable to *E. coli* J53 and EC600, with only data on J53 being shown here. S1-PFGE and Southern hybridisation performed on the parental and transconjugant of all 42 strains showed that *bla*_NDM_ was located on plasmids of six different sizes, with the ca 46 kb IncX3-type plasmid being the most dominant, accounting for 36 of the 42 conjugative plasmids. Two strains were found to harbour an IncB plasmid of ca 110 kb, and one strain each carried a plasmid of 54 kb (IncX3), 74 kb (IncFII), 97 kb (IncX1-IncN) and 130 kb (IncFIB) in size (Figure 1).

The ca 46 kb and ca 54 kb IncX3+type *bla*_NDM_-bearing plasmids have been commonly reported in clinical isolates, while they were rarely reported in carbapenem-resistant Enterobacteriaceae strains isolated from non-clinical settings. To confirm that these plasmids, recovered from *E. coli* of food origin, were genetically similar to those in human clinical isolates, one ca 46 kb plasmid and one ca 54 kb plasmid were recovered from the transconjugants and subjected to complete plasmid sequencing using both Illumina and Nanopore sequencing platforms. 

**Figure 2:**
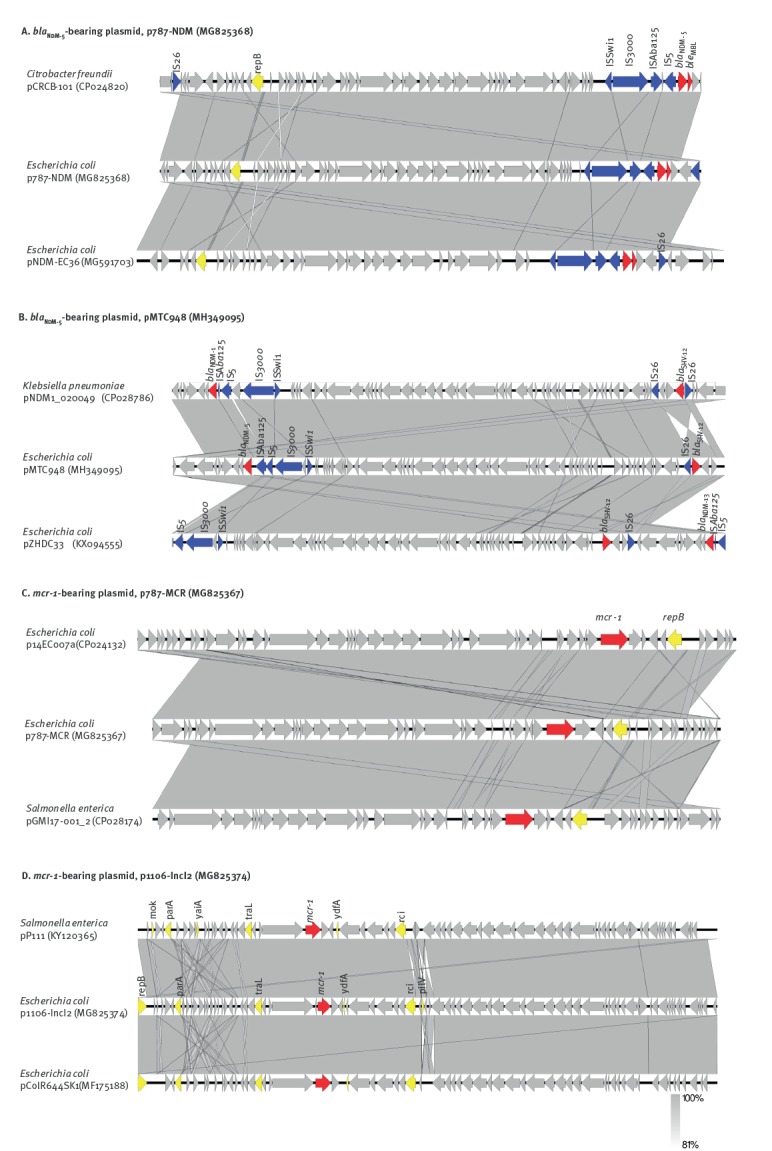
Schematic representation and alignment of complete sequence of *mcr-1*-bearing and *bla*_NDM_-bearing plasmids with homologous plasmids in GenBank, Shenzhen, 2015–2017*

The complete map of the *bla*_NDM-5_-bearing plasmid from transconjugant *E. coli* MTC787, designated as pMTC787, was 46,161 bp in size with a GC content of 46.7%; it was confirmed to belong to an IncX3 replicon type. BLASTN analysis of this plasmid revealed that p787-NDM displayed 100% query coverage and 99% nucleotide identity with a plasmid pCRCB-101_1 (CP024820) carried by a *Citrobacter freundii* strain CRCB-101 isolated from open pus of a person in South Korea, and with plasmid pNDM-EC36 (MG591703) carried by an *E. coli* strain EC36 isolated in Henan, China (Figure 2A). Moreover, p787-NDM showed high homology (> 98% in both identity and coverage) to other similar IncX3, *bla*_NDM_-bearing plasmids reported in GenBank, such as plasmid pNDM_MGR194 from *K. pneumoniae* (NC_022740.1), plasmid pP855-NDM5 (MF547508.1) and plasmid pP788A-NDM5 (MF547507.1) from clinical *E. coli* strains and plasmid pNDM5-SSH006 (MTKV01000083.1) from *Salmonella Typhimurium* strain SSH006. 

Grey shading indicates homologies between the corresponding genetic loci on each plasmid. Arrows indicate coding sequences, with arrowheads indicating the direction of transcription. Red: antibiotic resistance-encoding genes; blue: mobile elements; yellow: replicon proteins; grey: maintenance/stability functioning genes or hypothetical proteins.

The complete sequence *bla*_NDM-5_-bearing plasmid from the transconjugant of *E. coli* strain 948, MTC948 was obtained and designated as pMTC948. It was confirmed as an IncX3 type with a size of 53,770 bp. Sequence analysis showed that it was highly homologous to plasmid pNDM1_020049 which is carried by a *Klebsiella pneumoniae* strain SCKP020049 isolated from a person in Sichuan, China and to plasmid pZHDC33 which is carried by an *E. coli* strain ZHDC33 isolated in Zhejiang, China. Plasmid pMTC948 aligned well with plasmid p787-NDM, with only an additional mobile element carrying IS*26*-*bla*_SHV-12_ (Figure 2B). This plasmid also displayed high homology (> 98% in both identity and coverage) with other IncX3, *bla*_NDM-1_-bearing plasmids reported in GenBank, such as plasmid pNDM-HN380 (NC_019162.1) from *K. pneumoniae*, plasmid pNDM-EcHK001 (NZ_CM008823.1) from *Enterobacter cloacae* strain EcHK001 and plasmid pYQ13500-NDM (KR059865.1) from *En. cloacae* strain EC-YQ13500.

### Genetic features of *mcr-1*-bearing and *bla*_NDM_-bearing plasmids in *Escherichia coli*

**Figure 3:**
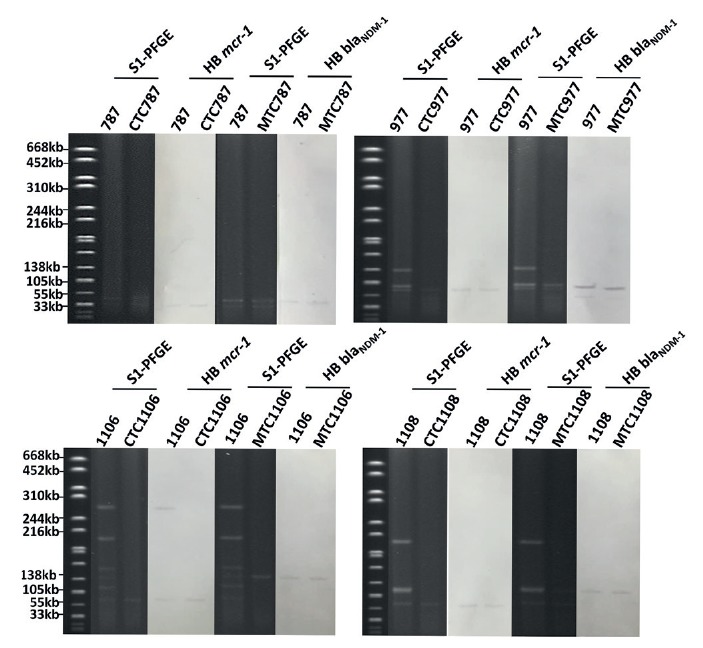
S1-PFGE and Southern hybridisation of *mcr-1* and *bla*_NDM-1_-bearing *Escherichia coli* strains and the corresponding transconjugants, Shenzhen, 2015–2017 (n = 2)

CTC: transconjugants selected with colistin; HB: Southern hybridisation; MTS: transconjugants selected with meropenem; PFGE: pulsed-field gel electrophoresis.

Among the 42 *E. coli* strains carrying *bla*_NDM_, five also carried *mcr-1* (Figure 1). Conjugation experiments were performed to assess the transferability of the colistin resistance phenotype; it could be successfully transferred to *E. coli* J53 from all strains except strain 1107, where the *mcr-1*-bearing plasmid was not conjugative and could not be transferred by transformation. S1-PFGE and Southern hybridisation were performed on strain 1107 and showed that the *mcr-1* gene was located on a ca 220 kb plasmid belonging to the InHI2 type. Two types of conjugative *mcr-1*-bearing plasmids could be recovered from strains 787, 1106 and 1108_,_ with three strains carrying a ca 60 kb IncI2 plasmid and one harbouring a ca 33 kb, IncX4 plasmid (Figure 1). Interestingly, during conjugation, both the *mcr-1*-bearing and the *bla*_NDM-1_-bearing plasmid in strains 787, 974 and 1108 could be simultaneously transferred to *E. coli* J53 under selection of meropenem, yet only *mcr-1*-bearing plasmids could be transferred to J53 under selection of colistin. The data suggested that even though the *mcr-1* and *bla*_NDM-1_ genes were located on different plasmids, each of these two plasmids could be transferred to other bacteria under meropenem selection pressure (Figure 3).

Both the *mcr-1*-bearing and the *bla*_NDM_-bearing plasmids could be transmitted to *E. coli* J53 under selective pressure of meropenem from strains 787, 977 and 1108.

One ca 60 kb (IncI2) and one ca 33 kb (IncX4) *mcr-1*-bearing plasmid were recovered from the transconjugants and subjected to complete plasmid sequencing using both Illumina and Nanopore sequencing platforms. The complete map of the ca 30 kb *mcr-1*-bearing plasmid recovered from transconjugant *E. coli* CTC787, designated as pCTC787, was shown to belong to IncX4 type with a size of 33,301 bp. It aligned well (> 99% in both identity and coverage) with several previously reported plasmids such as p14EC007a (CP024132), which was carried by an *E. coli* strain 14EC007 isolated from clinical patients in China, and pGMI17–001_2 (CP028174) which was carried by a *Salmonella* enterica strain CFSAN064033 isolated from a turkey sample in Germany (Figure 2C). The complete sequence of a ca 60 kb *mcr-1*-bearing conjugative plasmids, recovered from the transconjugant *E. coli* CTC1106 strain was shown to belong to the IncI2 type with a size of 60,960 bp and a GC content of 42.3%. It was designated as p1106-IncI2. BLASTN analysis revealed that it exhibited the highest homology with pP111, carried by a *Salmonella enterica* (KY120365) strain P111 isolated in Taiwan, and with pColR644SK1, carried by an *E. coli* strain ColR644SK1 (MF175188) isolated in Switzerland. It also exhibited high homology (> 99% in both identity and coverage) with pHNSHP45 (KP347127) and other plasmids reported previously [25,26]. Similar to other IncI2 type plasmids, p1106-IncI2 contained genes and proteins responsible for replication (*repB*), plasmid stability (*mok*), partitioning (*parA*), plasmid transferability (*traL*), plasmid-borne site-specific recombinase (*rci*) and pilus-encoding loci (*pilV*) (Figure 2D).

## Discussion

Mobile carbapenemase genes have emerged in clinical isolates but are rare in isolates from animals. In China, the first *bla*_NDM-1_ gene was reported in an *E. coli* strain (HK-01) in 2011 [8] and has since disseminated extensively in clinical settings [27-29]. The *bla*_NDM-1_ gene has mainly been reported on a ca 46kb and 54kb IncX3-type conjugative plasmid in China [9,29,30]. Although such a gene has since been reported in non-clinical bacterial isolates of different origins, such as animals and the environment, it was often carried by plasmids of various types mainly through insertion of the *bla*_NDM-1_-bearing mobile element into the backbone of different plasmids in bacterial strains of the same origin. These data appear to imply that bacterial strains, in particular *E. coli*, that commonly reside in animals and the farm environment might not be adaptable to the IncX3 type plasmids. Recently, Wang Y et al. reported the prevalence of NDM and MCR-1-producing Enterobacteriaceae strains in poultry and the farm environment, and that the prevalence of *bla*_NDM-1_ in these strains has increased sharply [19]. *E. coli* isolates carrying an IncX3 *bla*_NDM_-bearing plasmid were found to be prevalent in Enterobacteriaceae strains isolated from the poultry production chain [19]. Wang R et al. also reported the co-existence of *mcr-1* and different *bla*_NDM-1_ variants in *E. coli* and *K. pneumoniae* isolates originating from broiler, swine and cattle samples [31]. These finding suggest that co-transmission of *bla*_NDM_ and the already prevalent *mcr-1* gene has occurred among Enterobacteriaceae in animals, leading to selection of strains carrying both *bla*_NDM-1_ and *mcr-1* which are a risk to human health. This risk will become imminent if Enterobacteriaceae strains carrying both resistance genes become prevalent in our food products. However, isolation of *E. coli* carrying both the *bla*_NDM-1_ and *mcr-1* gene, in particular carriage of *bla*_NDM-1_ by the IncX3-type plasmid in *E. coli* strains of food origin, has not been reported previously.

We have been conducting comprehensive surveillance of multidrug-resistant *E. coli* in food products in China in the past few years. *E. coli* strains of food origin were shown in our study to carry *mcr-1* at an increasing rate, whereas the *bla*_NDM-1_ gene was rarely detected. In this study, we have shown that the *bla*_NDM-_positive isolates were increasingly detectable over time, with a prevalence rate of 0.3% and 1% in 2015 and 2016, respectively, rising sharply to 17% in 2017. This sharp increase in the prevalence of *bla*_NDM-_bearing *E. coli* strains in food products was mainly due to transmission of a ca 46kb IncX3 plasmid commonly harboured by clinical Enterobacteriaceae isolates. In addition, the increasing prevalence of *E. coli* strains carrying *bla*_NDM_ implies emergence of *E. coli* strains that carry both *bla*_NDM_ and *mcr-1*. In this work, all three commonly reported *mcr-1*-bearing plasmids were able to co-exist in a single *E. coli* strain. In addition to the highly prevalent IncX3 *bla*_NDM-1_-bearing plasmid, another five plasmid types were also reported in *E. coli* of food origin. Furthermore, all *bla*_NDM_-bearing plasmids reported in our study were conjugative, implying that they were transmissible to other *E. coli* strains of food origin. The increasing prevalence of Enterobacteriaceae strains carrying *bla*_NDM_ and *mcr-1* in food products will lead to increased colonisation of the human gastrointestinal tract with these Enterobacteriaceae strains, a phenomenon that has been associated with the high prevalence of drug-resistant infections in clinical settings [32]. Importantly, *E. coli* carrying *bla*_NDM-1_ or *bla*_NDM-1_ /*mcr-1* could be detected in pork, beef and even shrimp, suggesting that these strains have already widely disseminated to different food products. An increasing rate of food-borne infections caused by such strains would be a serious concern. Further surveillance of *bla*_NDM-1_ and *mcr-1* in other food products is warranted.

## *Erratum

After publication, typographical errors were corrected in the title of Figure 2. These changes were made on 3 April 2019.

## Supplementary Data

691000 Supplement
