# Erratum for Euro Surveill. 2019;24(13)

**DOI:** 10.2807/1560-7917.ES.2018.24.14.190404e

**Published:** 2019-04-04

**Authors:** Eurosurveillance editorial team

**Affiliations:** 1 European Centre for Disease Prevention and Control (ECDC), Stockholm, Sweden

In the article ‘Increased prevalence of Escherichia coli strains from food carrying blaNDM and mcr-1-bearing plasmids that structurally resemble those of clinical strains, China, 2015 to 2017, published on 28 March 2019, typographical errors were corrected in the title of Figure 2 after publication. These changes were made on 3 April 2019.

