# Supplementary material for: Increased prevalence of Escherichia coli strains from food carrying bla NDM and mcr-1-bearing plasmids that structurally resemble those of clinical strains, China, 2015 to 2017
Source: Euro Surveill. 2019 Mar 28;24(13):1800113. doi: 10.2807/1560-7917.ES.2019.24.13.1800113 (PMC6446510; doi:10.2807/1560-7917.ES.2019.24.13.1800113)
Supplement: Supplement [file 18-00113_Supplement_CHEN.pdf]

This supplementary material is hosted by Eurosurveillance as supporting information alongside the article [Alarming increase in prevalence of foodborne *E. coli* strains carrying *bla*$_{NDM}$ and *mcr-1*-bearing plasmids that structurally resemble those of clinical strains] on behalf of the authors who remain responsible for the accuracy and appropriateness of the content. The same standards for ethics, copyright, attributions and permissions as for the article apply. Eurosurveillance is not responsible for the maintenance of any links or email addresses provided therein

Supplementary materials

Alarming increase in prevalence of foodborne *E. coli* strains carrying *bla*$_{NDM}$ and *mcr-1*-bearing plasmids that structurally resemble those of clinical strains

Keywords: *bla*$_{NDM}$, *mcr-1*, Foodborne *E. coli*, Plasmid, Resistance

Supplementary methods:

Bacterial isolation

Each food sample was subjected to *E. coli* isolation using three different isolation methods. Firstly, contamination of extended spectrum beta-lactamase (ESBL)-producing *E. coli* was surveyed in different food samples. To do that, 25 g of each sample were placed into a sterile homogeneous bag containing 50 mL of sterilised saline. After homogenisation for a few minutes, a loopful of filtered saline was spread onto the MacConkey agar plates supplemented with 2 µg/mL cefotaxime. Secondly, contamination of carbapenem-resistant *E. coli* was surveyed. Food samples were prepared in the same way as above except that a loopful of food suspension was spread onto the MacConkey agar plates supplemented with 0.5 µg/mL meropenem. Thirdly, additional carbapenem-resistant *E. coli* were recovered using an isolation method for *Salmonella* isolation, which has been recently observed in our lab. In brief, 25 g of each sample were placed into a sterile homogeneous bag containing 50 mL of sterilised saline. 1 mL of homogenate was transferred to Lactose broth and incubated at 42 °C for 12–16 h. One mL each of pre-enriched broth was transferred to tetrathionate broth (TT broth) with brilliant green (Becton Dickinson) and Rappaport-Vassiliadis broth (RV broth), respectively, and incubated overnight at 37 °C. A loopful of the culture was inoculated onto the XLT4 agar plate supplemented with 0.5 µg/mL meropenem. Following incubation at 37 °C for 16 h, two or three suspective *E. coli* colonies were purified on MacConkey agar plates. All *E. coli* isolates were further confirmed by MALDI-TOF MS (MicroFlex LT mass spectrometer, Bruker Daltonics) and API20E test strip (BioMerieux, Inc).

Conjugation, *Xba*I-PFGE, S1-PFGE and Southern hybridisation

A filter mating experiment was carried out to study the transferability of the resistance phenotypes of the test strains. Overnight cultures of donors (*E. coli*) and recipients (sodium azide-resistant *E. coli* J53) were mixed together in a donor/recipient ratio of 4:1, collected on a filter and the filter membrane was

incubated on blood agar medium without antibiotic. To select transconjugants carrying the *bla*$_{NDM}$ gene, the donor/recipient mixture was suspended and spread onto MacConkey Agar containing meropenem (1 µg/mL) and sodium azide (200 µg/mL). Transconjugants that grew on such plate were assessed for the presence of *bla*$_{NDM}$ gene by PCR. To select strains harbouring the *mcr-1* gene, the donor/recipient mixture was spread onto on Eosin Methylene Blue Agar containing sodium azide (100 µg/mL) and colistin (2 µg/mL). Genetic identity of transconjugants that grew on such plates was verified by PCR targeting the *mcr-1* gene. The genetic relatedness of the transconjugants was determined by PFGE in the CHEF-MAPPER System (Bio-Rad) upon *Xba*I digestion. Cluster analysis of PFGE patterns was performed by the BioNumerics (Applied Maths) system. Genomic DNA of the *S. Braenderup* H9812 strain digested with *Xba*I was used as reference. S1 nuclease-PFGE was performed to characterise the plasmid profiles. The genetic location of *bla*$_{NDM}$ in all transconjugants, and that of *bla*$_{NDM}$ and *mcr-1* in transconjugants positive for *mcr-1*, was identified by Southern hybridisation, using a digoxigenin-labelled probe with the DIG-High Prime DNA Labelling and Detection Starter Kit II (Roche Diagnostics).

Supplementary Table S1. Numbers of different food samples purchased and numbers of *E. coli* isolated recovered from these food samples.

Year	No. of pork samples (No. of E. coli isolates)	No. of beef samples (No. of E. coli isolates)	No. of chicken samples (No. of E. coli isolates)	No. of shrimp samples (No. of E. coli isolates)	No. food samples from wet market (No. of E. coli isolates)	No. food samples from supermarket (No. of E. coli isolates)	Total
2015	432 (248)	72 (33)	130 (97)	113 (7)	496 (283)	251 (102)	747 (385)
2016	530 (319)	115 (61)	196 (168)	178 (22)	645 (405)	374 (165)	1019 (570)
2017	220 (143)	43 (13)	57 (50)	51 (5)	227 (137)	144 (74)	371 (211)
Total	1182 (710)	230 (107)	383 (315)	342 (34)	1368 (825)	769 (341)	2137 (1166)

Supplementary Table S2. Resistance rate, MIC_{50} and MIC_{90} of foodborne *E. coli* strains.

Antibiotics	Overall (n=1166)			Strains resistant to colistin (n=390)			Strains resistant to meropenem (n=42)		
	Resistant rate (%)	MIC_{50} (μg/ml)	MIC_{90} (μg/ml)	Resistant rate (%)	MIC_{50} (μg/ml)	MIC_{90} (μg/ml)	Resistant rate (%)	MIC_{50} (μg/ml)	MIC_{90} (μg/ml)
AMK	3	2	8	5	4	8	2	2	2
CTX	100	≥32	≥32	100	≥32	≥32	100	≥32	≥32
CIP	59	8	≥32	65	8	≥32	57	4	≥32
KAN	64	≥256	≥256	77	≥256	≥256	52	≥256	≥256
CRO	100	≥32	≥32	100	≥32	≥32	100	≥32	≥32
TET	95	≥64	≥64	96	≥64	≥64	91	≥64	≥64
CHL	86	≥128	≥128	94	≥128	≥128	81	≥128	≥128
NAL	82	≥128	≥128	91	≥128	≥128	79	≥128	≥128
AMP	100	≥128	≥128	100	≥128	≥128	100	≥128	≥128
SXT	97	≥64	≥64	99	≥64	≥64	93	≥64	≥64
CAZ/AVB	4	0.12	0.25	2	0.12	0.25	100	32	≥128
MRP	4	0.03	0.06	2	0.03	0.12	100	≥32	≥32
CLS	33	1	8	100	8	8	19	1	8
TIG	0	0.25	1	0	0.25	1	0	0.25	0.5

AMK, amikacin; CTX, cefotaxime; CIP, ciprofloxacin; KAN, kanamycin; CRO, ceftriaxone; TET, tetracycline; CHL, chloramphenicol; NAL, nalidixic acid; AMP, ampicillin; SXT, sulfamethoxazole/trimethoprim; CAZ/AVB: ceftazidime/Avibactam; MRP: meropenem; CLS: colistin; TIG, tigecycline.
